# Early discharge of first-time parents and their newborn: A scoping review

**DOI:** 10.18332/ejm/140792

**Published:** 2021-10-11

**Authors:** Victoria Lindblad, Pernille S. Gaardsted, Dorte Melgaard

**Affiliations:** 1 Department of Gynecology, Pregnancy and Childbirth, North Denmark Regional Hospital, Hjørring, Denmark; 2 Medical Library, Aalborg University Hospital, Aalborg, Denmark; 3 Center for Clinical Research, North Denmark Regional Hospital, Hjørring, Denmark; 4 Department of Clinical Medicine, Aalborg University, Aalborg, Denmark

**Keywords:** primiparity, length of stay, postnatal care, infant, early discharge

## Abstract

**INTRODUCTION:**

This scoping review aims to identify the evidence and the factors influencing the outcomes of early discharge of both healthy first-time mothers and newborns.

**METHODS:**

Systematic searches were conducted using four databases up to February 2021, and a search for grey literature was performed. A total of 2030 articles were identified and reduced to 13 articles, and one article was added through chain search in reference lists. The aims of the identified studies, the methodology, participants, inclusion and exclusion criteria, and the setting, context, and findings are summarized.

**RESULTS:**

A total of 14 studies were included. A thematic analysis identified the following factors influencing the outcomes of discharge within 24 hours after birth: parental education in pregnancy, perinatal information before discharge, sources of support, and follow-up strategies after discharge. Also, the analysis identified outcomes such as breastfeeding, parents' experience and readmission of the newborn that may be influenced when first-time parents are discharged within 24 hours after birth. Findings in this review highlight the importance of identifying factors and outcomes related to early discharge. However, because of the heterogeneity in methodology, terminology and assessment procedures used in the retrieved articles, the generalization of study results is limited.

**CONCLUSIONS:**

A gap in the literature about the outcomes of discharge within 24 hours after birth has been identified. Future studies with strong evidence are needed, defining criteria, context, and intervention.

## INTRODUCTION

Discharge within 24 hours after birth is not unusual for both healthy first-time mothers and newborns. A study on the length of stay after birth, including 30 low-income and middle-income countries, found that 28% of first-time mothers were discharged within 24 hours after a vaginal birth^1^. Furthermore, in Western countries, the length of stay after birth in a facility care unit has steadily decreased since the 1950s^2,3^. In addition, a study from Denmark from 2017, including 1202 first-time mothers, found that 9.1% of first-time mothers were discharged within 12 hours after birth^4^. Since 2018, healthy first-time mothers with uncomplicated births have been discharged within four hours after birth, as standard procedure at three larger maternity wards in Denmark. Therefore, the new tendency in Denmark leads to the interest of what evidence exists about the early discharge of first-time mothers. International recommendations on early discharge strategies for both healthy first-time mothers and newborns differ regarding the time of discharge after birth, the criteria for early discharge, and the follow-up strategies. The purpose of the recommendations is to prevent maternal and neonatal morbidity and mortality and prevent readmission^5-9^. For this reason, the United States government introduced the ‘Newborns’ and Mothers’ Health Protection Act’ in 1996, based on the recommendations of the American Academy of Pediatrics and the American College of Obstetricians and Gynecologists^10,11^. The law ensures coverage of a hospital stay for 48 hours following vaginal birth for parents with a group health plan or individual health insurance policy^10^. However, the World Health Organization (WHO) recommends staying in a healthcare facility for at least 24 hours after an uncomplicated birth^7^. The Spanish Association of Pediatrics recommends discharge from the hospital after 48 hours after birth for healthy newborns^5^. Furthermore, national guidelines from the United Kingdom, Denmark and Norway recommend that the time for discharge is based on discussion and agreement between the healthcare providers and the parents^6,8,9^. The overall criteria for discharge 24 hours after birth, recommended by WHO, are that the mother’s bleeding is controlled, the mother and newborn must have no signs of infection, and the baby should be breastfeeding well^7^. All the guidelines describe physical and psychosocial risk factors for the mother and newborn that must be considered by the healthcare professionals before early discharge after birth^5-9^, for example, signs of pre-eclampsia, postpartum hemorrhage, domestic abuse, and signs of neonatal jaundice^5-9^. Furthermore, the recommendations for follow-up strategies after discharge within 24 hours after birth differ. The recommendations include a home visit within 24 hours after birth, a pediatric check-up three to four days after birth, or a home visit by a midwife on the second or third day after birth^5-9^.

Reviews on early discharge after birth report factors that can influence the outcomes of early discharge of both healthy mothers and newborns. The influencing factors found in the reviews include the criteria for early discharge, parental education in pregnancy, information before discharge, infant feeding, follow-up strategies, and availability of support from family and health professionals after discharge^12-20^. The outcomes of early discharge examined in the reviews included postpartum hemorrhage, infection, breastfeeding, postpartum depression, neonatal dehydration or malnutrition, the father’s anxiety about caring for the newborn after discharge, and the parents' satisfaction with early discharge^12,20^. However, the definition of early discharge in the reviews varies from 6 to 96 hours after birth^12,20^. Early discharge for first-time parents is a broad subject, and a scoping review provides the opportunity to explore the extent and nature of the existing literature^21^. Our preliminary literature search showed that most studies define early discharge as discharge within 24 hours. However, early discharge was in some studies defined as within 8 hours, 17 hours, or the day of birth. To ensure that we identified all studies that addressed early discharge, we chose 24 hours as the inclusion criterion. The purpose of this scoping review is to aid maternity wards in developing an early discharge policy. Therefore, this scoping review aims to identify the evidence of: 1) factors that influence firsttime parents' decision or readiness to be discharged within 24 hours, and 2) outcomes for healthy first-time parents and healthy newborns who were discharged within 24 hours after birth and which factors influence these outcomes.

## METHODS

A scoping review provides the opportunity for summarizing the existing research in a field where the literature is heterogeneous in terms of the study design, theoretical framework, and outcome measurement^21^. This was the case for the outcomes and the factors influencing the outcomes of early discharge of healthy first-time parents. The PRISMA-ScR; Checklist and Explanation was used as a guide in structuring this article^22^. Furthermore, the Levac et al.^21^ methodological framework, with a six-step approach for conducting a scoping review, was used to guide the systematic literature search of this scoping review.

### Research questions

The research questions included: ‘What evidence exists about factors that influence first-time parents' decision or readiness to be discharged within 24 hours?’ and ‘What evidence exists about outcomes and factors that influence the outcomes of discharge within 24 hours after birth for healthy first-time parents and healthy newborns?’.

### Inclusion criteria

The authors conducted a comprehensive systematic literature search to identify relevant articles. The inclusion criteria were first-time parents and healthy mothers with an uncomplicated vaginal birth, healthy infants defined by the primary studies’ authors, and infants born at a gestation age between 37 to 42 weeks. We included first-time parents discharged within 24 hours after birth or described as discharged within one day after birth, compared to discharge later than 24 hours after birth. If a study included both first-time and multiparous parents, a clear distinction between the results for first-time and multiparous parents was required for inclusion. Also, a clear distinction between the results for discharge before or after 24 hours after birth, and a distinction between the results for vaginal birth and caesarean section were required for inclusion. Quantitative, qualitative, and mixed methods types of studies were also included. Study designs as metaanalysis, reviews, experimental and observational study were included. Conference abstracts, dissertations, books, essays, editorials, commentaries, and audio accounts were excluded. Articles written in English, Danish, Norwegian, and Swedish were included.

Pubmed, Cinahl (with full text), EMBASE (Ovid), and Scopus were systematically searched from the inception date of each database to 8 October 2019. Keywords in the search included: first-time parents (e.g. primipara, parents, or mother), first child (e.g. infant, or neonatal) and length of stay after birth (e.g. early discharge, labor, ambulatory care, or outpatient). The search included synonyms and variations of the keywords combined with Boolean operators. A combination of search techniques was used, including controlled vocabulary thesaurus terms (e.g. medical subject headings – MeSH, and Cinahl headings) and a free-text search of all the synonyms and variations of the keywords were used. Furthermore, to identify any grey literature, a search in Google Scholar was performed. Finally, the searches were supplemented with forward and backward chain search in citations and reference lists from the identified literature and relevant guidelines. The search strategies from all databases are shown in the Supplementary file.

### Study selection

The authors independently screened the records in Covidence^®^ according to the inclusion criteria^23^. Disagreements were discussed and resolved by consensus of the authors. An updated search was performed on 22 February 2021, based on the search strategy described above.

### Data reporting

The data were charted in stage four, according to Levac et al., and a descriptive numeric summary and a thematic analysis guided by the research question was provided. Each study was narratively described. Authors VL and DM collaborated thoroughly in extracting the data from the identified studies.

## RESULTS

### Search results

**Figure 1:**
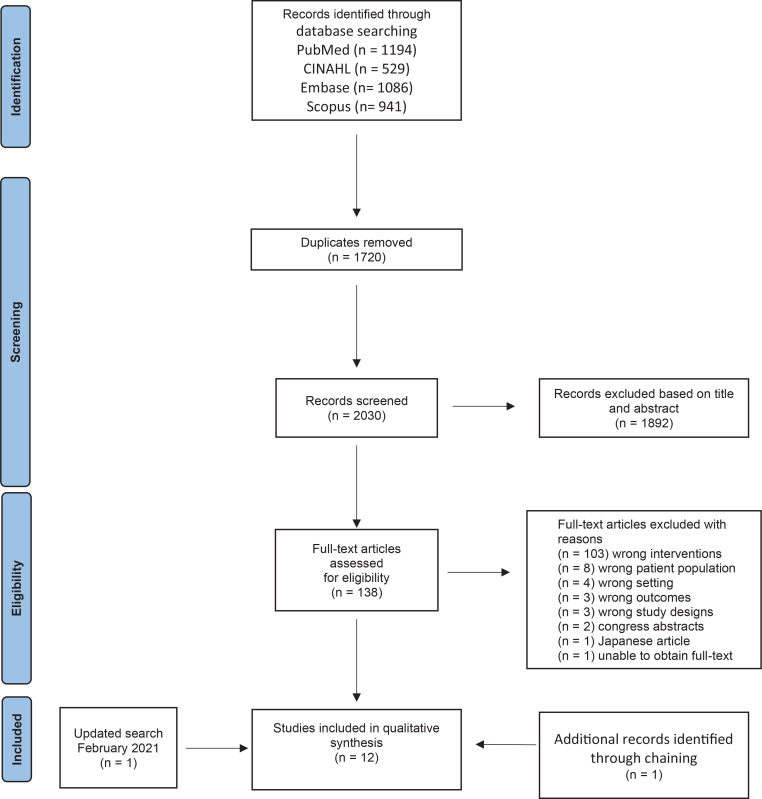
Prisma 2009 Flow Diagram

Based on the search strategy, 3750 citations were identified. After removing duplicates, 2030 studies were screened independently, by authors VL and PSG, based on title and abstract, and 1892 studies were irrelevant. Authors VL and DM screened independently a total of 138 studies based on full text. A total of 126 studies were excluded. The updated search identified a total of 366 studies, and one new study was included. Thus, a total of 13 studies were included, and one article was added through chain search in reference lists, as shown in the PRISMA flow diagram (Figure 1).

As illustrated in Table 1, 14 studies fulfilled the inclusion criteria in this scoping review. The studies were published from 1987 to 2020. Six of the studies used a qualitative approach, and seven studies used a quantitative approach. One study used a mixed-methods design. Among the quantitative studies, three of the seven quantitative studies used questionnaires, two used surveys, one was a retrospective cohort study, and one used a Q-technique as study design. The studies included in this scoping review took place in the United States of America (n=5), Denmark (n=4), Sweden (n=2), Australia (n=2), and Norway (n=1).

**Table 1:** Summary of data of the included studies

*Author, year and country*	*Methodology*	*Aims of study*	*Participants, inclusion, and exclusion criteria*	*Setting and context*	*Findings*
James et al.^33^ 2020 Australia	Qualitative Semistructured interview	First time-mothers experience of establishing breastfeeding following early discharge.	Participants (n=12) Inclusion criteria: Primiparous; Normal vaginal birth; Term infant; Antenatal intention to breastfeed; Spoke English.	Social media Facebook Discharge within 24 hours after birth.	Three themes were identified as heavily embedded in the mothers breastfeeding experience: Self-efficacy influences the mother’s readiness and motivation to breastfeed and be discharged home early; Support, social- and professional support important for the mothers breastfeeding selfefficacy; Sustainability was enhanced by timely and community-based breastfeeding supports and reliable online resources.
Johansson et al.^28^ 2019 Sweden	Mixed method Questionnaire with open and closed questions	Mothers’ experiences in relation to a new postnatal home-based model of midwifery care.	Participants (n=180) First-time mothers (n=10) Inclusion criteria: Swedish speaking; Healthy mothers; Uncomplicated pregnancy; Uncomplicated vaginal birth; Healthy term newborn. Exclusion criteria: Not specified.	University hospital Discharge within 24 hours after birth Before discharge: The midwife and the pediatrics examined the newborn; Health examination of the mother; Information package to the parents regarding the mothers and newborn’s health and breastfeeding. Follow-up strategy in the first week after birth: Daily telephone contact and/or home visit 24-hours post-birth; Pediatric examination in hospital; 24-hour hotline for the parents.	90% of first-time mothers felt very much or much safe when being discharged.
Feenstra et al.^31^ 2019 Denmark	Qualitative Hermeneutic interview	Mothers experience of the time from birth to being discharged after readmission with their newborn.	Participants (n=7) First-time mothers (n=4) Inclusion criteria: Uncomplicated pregnancy and birth; Physically and mentally healthy mothers; Infants born between gestation 37–42 weeks; Healthy newborns. Exclusion criteria: Mothers that did not speak Danish, Swedish, Norwegian, or English.	University Hospital Discharged within 24 hours after birth. Follow-up strategy: Outpatient’s clinics available for check-ups; Telephone and online consultations accessible around the clock; Home visits available; Check-up at the outpatient clinic 48–72 hours after birth; The health visitor from the municipality visits on day 4/5 and 2 weeks after birth.	Before early discharge: Enjoyed being alone as a family in the room. After discharge: Appreciated the calmness at home; Thought it was hard, needed a lot of help with breastfeeding; Sad and cried, had a lot of pain, felt insecure.
Aune et al.^29^ 2018 Norway	Quantitative Questionnaire	Examine the difference between an intervention and control group. The objective was: To assess the length of stay in the hospital. To investigate the possible positive influences of a home visit by a midwife, two to six days after birth	Participants (n=183) First-time mothers (n=102) Inclusion criteria: Healthy newborn at term; Speaking Norwegian. Exclusion criteria: Not specified.	University Hospital and public health clinic Discharge within 24 hours after birth. In pregnancy: The midwife offered a home visit to the family after birth. Follow-up strategy: One day after birth, the midwife made an appointment for a home visit, two to six days after birth; The home visit included e.g. guidance in breastfeeding and baby care, a physiological examination of the baby and mother, and a dialogue about the births and the mother’s birth experience; All mothers had a home visit by the public health nurse 7–14 days after birth.	4 % of the first-time mothers left hospital within 24 hours after birth. The first-time mothers left hospital earlier in the intervention group than in the control group. A visit by the midwife seems to have a positive health-promoting influence on first-time mothers’ confidence and ability to handle the care of their newborn and on perceived support, predictability, and continuity of midwifery care.
Feenstra et al.^26^ 2018 Denmark	Qualitative Hermeneutic interviews	How new fathers experience early discharge after birth and readmission of their newborn in relation to their role and involvement as a father.	Participants (n=6) First-time fathers (n=3) Inclusion criteria: Mother with uncomplicated pregnancy and birth; Healthy newborns at term gestation 37–42 weeks. Exclusion criteria: Fathers who did not speak Danish, Swedish, English, or Norwegian.	University Hospital Discharge within 24 hours after birth. Follow-up strategy: Outpatient clinics available for check-ups; Telephone and online consultations accessible around the clock; Home visits available; The health visitor from the municipality contacts the family within the first week at home.	After birth before early discharge: Experienced busy midwives, lot of information, and waiting time; Felt confident going home; Felt good about being discharged early. After early discharge: Felt unsecure, worried, powerless; Unanswered questions, felt unprepared
Danbjorg et al.^30^ 2015 Denmark	Qualitative Participatory design	How postnatal parents experienced the use of telemedicine following early discharge from hospital by investigating if they consider that their postnatal needs are met, and whether they experience a sense of security and parental self-efficacy.	Participants (n=42) First-time mothers (n=7) Inclusion criteria: Discharge within 24 hours after birth; Uncomplicated pregnancy and birth; Physically and mentally healthy adults; Term healthy newborns. Exclusion criteria: Mothers not speaking Danish.	Regional hospital Discharge within 24 hours after birth. Follow-up strategy: App with 1) online chat (text messages, photos, and videos) with answers within four hours; 2) knowledgebase and issued messages automatically every 12 hours from the time of birth.	First-time parents viewed the app: As a lifeline; As a means for informing guiding following early discharge. This app shows potential for: Enhancing self-efficacy; Enhancing postnatal sense of security.
Danbjorg et al.^27^ 2014 Denmark	Qualitative Participatory design	Identify the needs for nursing support of new parents and their newborns during the first seven days after birth, based on the parents' experiences with early postnatal discharge.	Participants (n=37) Parents (n=19) Ten first-time parents (7 mothers, 3 fathers) Healthcare professionals (n=18) Inclusion criteria: Discharge within 24 hours after birth. Exclusion criteria: Complicated births; Parents not speaking Danish.	Regional hospital Discharged within 24 hours after birth.	Before early discharge: Early discharge generated a pressure; Feelings of being ‘kicked out’; Feelings of insecurity. After early discharge: Dependent on family and friends; Need for available healthcare professionals around the clock; Had questions regarding care of the newborn.
Johansson et al.^37^ 2010 Sweden	Qualitative Explorative study focus-group interviews	First-time parents' experiences of early discharge after birth and home-based postnatal care.	First-time parents (n=21) Mothers (n=11) Fathers (n=10) Inclusion criteria: First-time parents; Healthy mothers; Normal pregnancy and birth; Healthy and full-term newborn; Swedish speaking couples; Discharge immediately from the delivery ward and within 24 hours and without further postpartum care at the hospital; Living in Uppsala community; Parents cohabiting at the time of birth. Exclusion criteria: Not specified.	University Hospital Discharged between 6 and 17 hours after birth. Follow-up strategy: Specially trained midwives offer home visits as often as the families need until the fourth day after birth; Telephone call the day after discharge; The fifth day a second pediatric examination and a metabolic screen at the hospital.	Before early discharge after birth: Wanted to go home as soon as possible, home is best, wanted to sleep in their own bed; Approximately half of the parents had planned to stay, but changed their mind, because they felt good about it. After early discharge: Father felt secure and in control at home; Felt safe and knew that they easily could get in contact with midwives around the clock; Uncertain about care for the baby, felt free and independent, feelings of shared responsibility between the parents; Good that the midwives initiated the contact after discharge and the midwives gave the parents confidence at the home visit.
Danielsen et al.^34^ 2000 USA	Quantitative Retrospective cohort study	To assess the impact of very early discharge (defined as discharge on the day of birth) on the risk of newborn readmission during the neonatal period.	Births (n=1214545) from 1992 to 1995 Inclusion criteria: Healthy newborn; Vaginal birth; Routinely discharged to home. Exclusion criteria: Not specified.	Hospital Discharged on the day of birth.	Increased risk of readmission is associated with very early discharge (defined as discharge on the day of birth), OR=1.21.
Moran et al.^35^ 1997 USA	Quantitative survey	Investigate predictors of mothers’ desire to receive more information.	Participants (n=1161) First-time parents (n=540) Inclusion criteria: Discharge with a live newborn. Exclusion criteria: Multiple gestation; Newborns admitted to a neonatal intensive care unit or transferred to another hospital after birth; Consistent missing survey information.	Hospitals (n=3): one urban one suburban, one urban and serving surrounding rural areas. Discharged within one day or less after birth.	Over three-fourths of first-time mothers wanted more information on at least one topic. They wanted information on exercise, diet, and nutrition; feelings of fatigue and resuming normal activities. First-time mothers with a short hospital stay after birth wanted information about significantly fewer self-care and baby care items than those with longer stay. First-time mothers who desired more information about two self-care topics were aged <25 years. First-time mothers with less than high school education were more likely to want more information on most baby care topics. First-time mothers with low levels of social support were more likely to want more information about a variety of topics. Prenatal education was unrelated to postpartum desire for more information.
Quinn et al.^32^ 1997 USA	Quantitative Descriptive two-group survey	Determine if the incidence of breastfeeding at 6–8 weeks after birth differs for first-time mothers who had a 48 hours length of stay versus a 24 hours length of stay after birth and what factors influenced the change from breastfeeding to bottle-feeding.	First-time mothers (n=101) Inclusion criteria: English speaking; Age 18–35 years; Telephone access; Vaginal birth; Healthy newborn. Exclusion criteria: If mother and newborn were separated.	Regional hospital Discharged within 24 hours after birth. Follow-up strategy: One home visit on day three after birth in the group that was discharged within 24 hours after birth; The group discharged within 48 hours after birth did not receive a home visit.	No difference in incidence of breastfeeding 6–8 weeks after birth. Factors influencing breastfeeding cessation were similar in the two groups. The factors were: Perception that the infant did not get enough milk; The mother returned to work or school; Ease of formula use.
Handfield and Bell^24^ 1995 Australia	Quantitative Questionnaire	Investigated the role of childbirth education in relation to making decisions about breastfeeding, pain medication and length of hospital stay after birth.	First-time mothers (n=59) Inclusion criteria: Attended classes in pregnancy. Exclusion criteria: Not specified.	Municipal hospital Discharged within 24 hours after birth. Antenatal classes by midwives: Five sessions, two hours in length; One session in early pregnancy; Four sessions in the third trimester; Postnatal issues are given four and a half hours of class time; The classes are not compulsory but highly recommended.	The information they received had minimal effect on the mother’s feelings about the appropriateness of a 24-hour stay after birth; Three mothers who had planned to stay longer than 24 hours changed their mind and were discharged within 24 hours, but they did not attribute the change in opinion to classes; 20 of the 31 mothers who had planned to be discharged within 24 hours changed their opinion to a longer stay; Educational strategies have failed to address the tendency of first-time mothers to postpone making decisions about the postnatal period, such as early discharge.
Martell et al.^36^ 1989 USA	Quantitative Q-technique to rank the mother’s priorities	Identify the information from the teaching protocol content that mothers in a maternity short-stay program thought was most important to them the first 3 days after birth	Participants (n=42) First-time parents (n=14) Inclusion criteria: Mothers had assistance at home for the first week. Exclusion criteria: Extreme age; Extreme gestation; Extreme length of labor.	Regional hospital Discharged between 6–8 hours after birth. Follow-up strategies: Nurses teaching mothers before discharge; Mother and newborn visits the outpatient clinic between 48 and 72 hours after birth.	First-time mothers ranked the items as follows (with the most important item first): 1) Warning signs 2) Infant care 3) Infant feeding formula 4) Infant breastfeeding 5) Uterine massage 6) Breast care if breastfeeding 7) Perineal care 8) Breast care if formulae 9) Comfort measures 10) Family changes 11) Involution 12) Rest 13) Bowel function 14) Sexuality
Lemmer^25^ 1987 USA	Quantitative Questionnaire	Impact early discharge has on the outcomes of first-time parents and their newborns. Research questions: 1) are the postpartum concerns of first-time mothers discharged within 24 hours after birth and the postpartum concerns of first-time mothers choosing later discharge different? 2) why do mothers choose early discharge?	42 first-time mothers (n=42) Inclusion criteria: Gravida 1, abortion 0; No history of infertility; Aged ≥20 years; Vaginally birth; Temperatures less than 38.0℃; No postpartum hemorrhage; Blood pressure less than 140/90 mmHg; Able to read and speak English; 38–41 weeks’ gestation; 2500–4500 g birth weight; Documented normal physical examination; Newborn stable temperature; Demonstrated ability to feed, i.e. suck and swallow mechanisms intact. Exclusion criteria: Not specified.	University hospital Discharged within 24 hours after birth. A home visit was made six to eight days after birth. Any abnormal findings were followed up by physical examination.	No differences in medical outcome in the two groups. No significant differences in maternal concerns. Factors motivating choice of early discharge: Comfort; Financial concerns; Recognition that a healthy mother and infant were as safe at home as in the hospital; More sources of support at home.

#### Definition of early discharge

Most of the included studies define early discharge as taking place within 24 hours after birth^24-33^. However, some of the studies use other definitions like discharge on the day of birth^34^, discharge within one day or less^35^, discharge six to eight hours after birth^36^, or six to 17 hours after birth^37^.

#### Definition of uncomplicated birth

The definition of an uncomplicated birth is not specified in most of the studies. In six studies, the criterion for early discharge is described as an uncomplicated pregnancy and birth, without further definition^27,30-33,37^. Other studies used criteria for early discharge that included all routinely discharged healthy newborns at term^29,34^ or all mothers discharged with a live newborn^35^. One study^25^ had the following specific criteria for early discharge:

- Gravida 1
- Abortion 0
- No history of infertility
- Age of the mother >20 years
- Vaginal birth
- Temperatures below 38.0℃
- No postpartum hemorrhage
- Blood pressure below 140/90 mmHg
- 38–41 weeks gestation
- 2500–4500 g weight at birth
- The newborn has a stable temperature and can feed, i.e. suck and swallow.

The factors that might influence the first-time parents' decision or readiness to be discharged within 24 hours after birth identified in this scoping review were parental education, social support, and follow-up strategies after discharge. The following four outcomes of early discharge were identified: the parents' sense of security, the parents' satisfaction with early discharge, breastfeeding, and neonatal readmission. In addition, five factors that might influence one or more of the outcomes were parental education in pregnancy, social support from relatives, follow-up strategies, availability of support from healthcare professionals, and the parents' readiness for discharge.

### Factors that influence the first-time parents' decision or readiness to be discharged within 24 hours after birth

A study found that first-time parents can be divided into two categories related to the parents' choice of early discharge after birth^37^. One group of parents had ‘decided beforehand’, and another group wanted to ‘take it as it comes’^37^.

#### Parental education

A descriptive study documented, that prenatal classes had minimal effect on the mothers’ readiness to be discharged from the hospital 24 hours after birth^24^. Of the 10 hours of classes, four and a half hours were focused on postnatal issues, but further details were not described^24^. Of the 59 mothers in the study, eight mothers changed their opinion from a preferred longer stay to being discharged 24 hours after birth. Only three of the mothers attributed their change in opinion to the information given in the prenatal class^24^. A total of 20 of the 59 mothers changed their opinion after birth, from wanting discharge within 24 hours after birth to wanting discharge later. However, 15 of these mothers attributed the change in opinion to the birth experience and not the prenatal education^24^. Lemmer^25^ found no difference in attendance in childbirth classes for first-time mothers who chose discharge before or after 24 hours after birth. In another study including 540 first-time mothers, 27.6% stayed in the hospital for less than one day^35^. Approximately 80% of all the first-time mothers in this study received prenatal education^35^. However, the proportion of first-time mothers discharged within one day and received prenatal education was not reported^35^. Furthermore, the content of prenatal education was not described^35^. Prenatal education seems to have little impact on the mothers’ readiness or decision to be discharged within 24 hours. The studies all lacked a detailed description of the content of parental education.

#### Support from relatives

A survey compared two groups of first-time parents who were discharged before or after 24 hours after birth^25^. The study found that the mothers in the short-stay group reported more sources of support than the group that chose to remain in the hospital for more than 24 hours after birth^25^. Furthermore, the social support came specifically from the mother’s husband, mother, and mother-inlaw^25^. This indicates that social support from the nearest relatives might positively influence the parents' readiness for discharge within 24 hours after birth.

#### Follow-up strategies

One study examined the mothers’ choice of the postpartum length of stay when the mothers were offered one home visit from a midwife between the second and sixth day after birth^29^. This study found that only 4% of first-time mothers left the hospital within 24 hours after birth^29^. In comparison, none in the control group, that did not receive an offer of a home visit by the midwife, left the hospital within 24 hours^29^. The home visit by the midwife included guidance in breastfeeding and about baby care, a physical examination of the mother and baby, and dialogue about the birth and the mother’s experience^29^. Therefore, it seems that a follow-up strategy with one home visit by a midwife between the second and the sixth day after birth was not enough to make the parents want to leave the hospital within 24 hours after birth.

### The first-time parents' sense of security when discharged within 24 hours after birth

A qualitative study including 10 first-time parents (7 mothers and 3 fathers) found that first-time parents were particularly dependent on their respective network of family and friends^27^. They expressed a sense of confidence and praise for their accessibility to family and friends when they needed assistance^27^. The availability and support from relatives had a positive effect on the parents' sense of security. On the other hand, two first-time mothers felt pressure from the health professionals at the hospital to be discharged within 24 hours after birth, which generated a sense of insecurity among the parents^27^. Therefore, if the parents do not feel ready for discharge, it might have a negative effect on the parents' sense of security after discharge. Another study of a new home-based model of midwifery care found that 9 out of 10 first-time mothers felt safe or very safe when being discharged within 24 hours after birth^28^. The home-based model included a systematic follow-up strategy with a pediatrician and midwife examining the newborn and a health examination of the mother before discharge^28^. Furthermore, an information package regarding the mother’s and newborn’s health and breastfeeding was handed out^28^. After discharge, the follow-up strategy was daily telephone contact during the first week and an offer of a home visit and a hospital visit^28^. In a qualitative study, one first-time father reported that they wanted a healthcare professional nearby to provide a sense of security, especially at night^27^. Another qualitative study found that first-time parents felt safe to be discharged within 24 hours after birth when they could easily contact midwives, day and night, and know that a midwife’s home visit was planned for the day after discharge^37^. Therefore, healthcare professionals’ availability and frequent contact with the parents are important to the parents' sense of security, and the parents must feel ready for discharge to feel secure.

### The parents' need for information in pregnancy and before discharge within 24 hours after birth

Two studies examined how the mothers prioritized their need for the information they received in pregnancy and before discharge from the hospital^35,36^. The first study found that first-time mothers discharged before 24 hours after birth were less likely to want information about feelings of fatigue, breastfeeding, bottle-feeding, night-feeding, and circumcision (males only) than those who stayed longer than 24 hours after birth^35^. First-time mothers under 25 years and discharged within 24 hours after birth were more likely to want information on self-care items, such as getting along with their husbands and the side effects of medication. Mothers with less than high school education were more likely to want more information on most baby care topics than mothers who graduated from high school or college^35^. The baby-care topics included changing, breastfeeding, their baby’s schedule, calming a crying baby, and recognizing illness^35^. First-time mothers discharged within 24 hours after birth with low levels of social support were more likely to want information about various topics. The topics included feelings of fatigue, resuming sexual activity, getting along with their husband, night-feeding, and their baby’s schedule^35^.

In the second study, 14 first-time mothers discharged within eight hours ranked the information they wanted after birth before being discharged^36^. Most mothers ranked information about identifying signs and symptoms of the newborns’ illness as the most important information before discharge^36^. The following important areas were signs and symptoms of maternal illness, what to do if a newborn was choking on mucus, and how to know if the newborn was getting enough milk^36^. Of medium importance was feeding and infant care, comfort measures, uterine massage and involution, rest, and breast care^36^. The resumption of intercourse and frequency of perineal pad change was ranked as least important for first-time mothers^36^. Subjects concerning a mother’s body change were consistently ranked lower than those related to infant care and feeding, and explanations of maternal body changes were almost consistently lower ranked than practical or ‘how-to’ information about the newborn^36^. The two studies show that the mothers need for information differs regarding discharge time after birth, education level, age, and amount of social support. A study based on focus group interviews and interviews with couples of 21 firsttime parents discharged between 6–17 hours after birth found that some parents felt uncertain about nursing and caring for the newborn^37^. However, some first-time parents did not have concerns about this^37^. A qualitative study including three first-time fathers discharged within 24 hours after birth reported that they felt it was nice to go home but felt unprepared^26^. One of the fathers wanted more information before he and the mother returned home from the maternity ward^26^. The details about parental education or information the parents received before discharge were not described in the latter two studies mentioned above. A study explored how mothers discharged within 24 hours experienced a follow-up strategy to access an app offering online chat with the midwife^30^. The mothers also had access to information material via the app^30^. Furthermore, the app automatically sent messages with information about signs of the baby’s well-being according to the baby’s age and information about breastfeeding^30^. One first-time mother found it easy to get an overview of the information and said, ‘I have read it all. I also look things up that I would not ask the nurse’^30^. The parents who used the app reported that the app provided timely information and guidance, and the parents felt that their needs for support after discharge were met^30^.

### The first-time parents' satisfaction with discharge within 24 hours after birth

A qualitative study, where early discharge was voluntary, found that first-time parents who were discharged within 24 hours after birth expressed positive feelings about freedom, self-reliance, shared responsibility, and independence^37^. However, there was also a feeling of ‘unreality’ of the newborn being there^37^. Another qualitative study found that home-based postnatal care is well accepted by first-time mothers leaving the hospital within 24 hours after birth^28^. The parents in the study were asked if they were interested in early discharge followed by home-based postnatal care^28^. Most of the first-time mothers had a positive postnatal care experience^28^. In a final study, the first-time mothers that chose to leave the hospital within 24 hours reported that a home visit significantly influenced their ability to handle the care of their newborn, benefited their mental health and their feeling of being recognized and supported^29^. The common denominator of the parents who were satisfied with early discharge in all the studies was that early discharge was voluntary, indicating that the parents' influence on the decision to be discharged early affects the parents' satisfaction of early discharge.

### The parents' feelings of stress when discharged within 24 hours after birth

First-time parents who were discharged within 24 hours with an app that send messages automatically made some first-time mothers feeling stressed when, for example, they received a message such as: ‘Now, your baby should have at least four full nappies a day’^30^. Another qualitative study including three first-time fathers discharged within 24 hours after birth reported that these fathers found the time from birth to discharge stressful and hectic^26^. The fathers found that the midwives were busy, the parents spend much time waiting for the midwives, and the amount of information the parents had to receive before discharge were all factors that contributed to the feelings of stress^26^.

### Breastfeeding incidence after discharge within 24 hours after birth

A descriptive survey including 101 first-time parents found no difference in breastfeeding six to eight weeks postpartum for mothers who had a 24- or 48-hour length of stay after birth^32^. The group that was discharged after 48 hours did not receive a home visit, and the parents discharged within 24 hours after birth received a home visit by a midwife on the third day after birth^32^. A qualitative study found that breastfeeding is described by first-time mothers who are discharged within 24 hours after birth as the ‘main thing’ and important, but challenging^37^. Some mothers felt breastfeeding was difficult, boring and took too long, but it became easier over time^37^. Through breastfeeding, mothers learned to trust themselves and their newborns. Breastfeeding was described as an interaction between the mother, the newborn, the father, the midwife, and the environment^37^. Another qualitative study, including 12 mothers, found that breastfeeding support with emotional reassurance, promotion of positive self-efficacy and accessible breastfeeding support after early discharge were important for a mother’s breastfeeding experience and sustaining breastfeeding after early discharge^33^.

### Readmission of the newborn after discharge within 24 hours after birth

A retrospective cohort study including 1214545 births found that newborns of first-time parents discharged on the day of the birth had a statistically significant higher risk (OR=1.21) of readmission than newborns discharged after a one- or two-night stay in the hospital^34^. Two studies reported that the parents experienced a lack of sleep, inexperience, and insecurity from discharge to neonatal readmission^26,31^. The lack of sleep and insecurities left the fathers feeling ill-prepared, and some felt powerless^26,31^. The mothers reported that they appreciated the calmness at home but ended up worrying, experiencing breastfeeding problems and being in pain^26,31^.

## DISCUSSION

The purpose of this scoping review was to give an overview of existing literature that can contribute to the development of policies at the maternity wards regarding postnatal care for first-time parents. Therefore, this scoping review aimed to identify the nature and extend of the existing evidence of what effect discharge within 24 hours has on first-time parents and their newborns. Furthermore, the aim was to identify evidence of factors that can influence the outcomes of early discharge of first-time parents.

Three factors that might influence the first-time parents' readiness or decision to be discharged within 24 hours after birth were examined in the studies identified in this scoping review. First, prenatal education was found to have minimal effect on the parents' readiness or decision to be discharged early. Second, social support from the nearest relatives was found to positively affect the parents' decision to leave the hospital early after discharge. Third, a followup strategy with one home visit by a midwife between the second and the sixth day did not make the parents want to leave the hospital within 24 hours after birth. Four primary outcomes were identified in this scoping review and included the parents' sense of security after discharge, the parents' satisfaction with early discharge, breastfeeding, and neonatal readmission. Factors that might influence one or more of the outcomes were parental education, social support from relatives, follow-up strategies, availability of support from healthcare professionals, and the parents' readiness for discharge.

### Factors that need to be considered when interpreting the results of the included studies

Overall, the studies were conducted in countries with different healthcare systems, which might influence the outcomes and make it difficult to compare the results. Thus, five studies were performed in the US and were conducted between 1987 and 1997. From 1996 the Newborns’ and Mothers’ Health Protection Act ensures a hospital stay if the mothers are covered by a group health plan or individual health insurance policy^10^. Between the years 1997 to 2000, approximately 15% of all people in the US did not have health insurance^38^. Seven of the studies were conducted in western countries and all after the year 2010. In Norway, Sweden and Denmark, the healthcare systems are tax-funded with free access to the healthcare system in pregnancy, birth, and postpartum^8,9,39^. The last two studies were performed in Australia in 1995 and 2020, and there has been free access to the healthcare system since 1986^40^. Moreover, the paid maternity leave differs substantially between the countries where the included studies were conducted. Thus, in 2016, mothers in Sweden had 35 weeks paid leave after birth^41^. In Norway, mothers had 45 weeks paid leave, and in Denmark, mothers had 27 weeks paid leave^41^. In contrast, Australian mothers had eight weeks paid leave, and in the US, mothers did not have any paid leave^41^. All the above factors must be taken into consideration when interpreting the results of the included studies.

Furthermore, the time for discharge varied in the included studies from six hours after birth to 24 hours after birth. The different discharge times might influence the parents' satisfaction with early discharge and the information they receive before discharge. It could also influence breastfeeding incidence since the mothers that stay 24 hours receive more guidance before discharge. Therefore, it could potentially affect the neonatal readmission rate. The criteria for early discharge in the studies is another factor to consider before comparing the results. The study by Lemmer^25^ was the only study in this review describing the criteria for early discharge in detail according to the obstetric factors. For example, it is well documented that a blood loss of over 500 mL after birth increases maternal morbidity and mortality and increases the risk of delayed breastfeeding initiation^42^.

### The parents' readiness to be discharged early after birth

Three studies identified in this scoping review concluded that parental education had minimal effect on the parents' readiness or decision to be discharged early^24,25,35^. However, the three studies were from 1987, 1995 and 1997, and more updated research is needed to explore if the educational programs today have improved regarding preparing the parents for early discharge. One of the studies showed that the birth experience had much more impact on the mother’s decision to stay longer than 24 hours after birth. We speculate that the mother’s birth experience still needs to be considered when deciding when the parents are discharged. In conclusion, there is a need for more and updated research on improving the parents' readiness for discharge within 24 hours.

### The parents' sense of security when discharged early after birth

The maternity wards follow-up strategies might impact the parents' feelings of safety when being offered early discharge after birth. Thus, Aune et al.^29^ found that parents offered as many home visits as the parents needed up to the fourth day after discharge, made nine out of ten mothers feel safe regarding early discharge. However, the study lacked a description of how many home visits the parents received after discharge. Furthermore, Johansson et al.^29^ found that only 4% of the first-time mothers chose to leave the hospital within 24 hours when they were offered one home visit, between the second and sixth day after discharge. Thus, a gap in the literature has been identified regarding the number and content of home visits the parents need to feel safe with early discharge.

The parents' need for parental education in pregnancy and information before discharge is also a factor that can influence the parents' sense of security. Three studies addressed the issue of prenatal education in pregnancy^24,25,35^, however, the studies lacked detailed descriptions that addressed this issue. Therefore, the lack of a detailed description of the content and form of the education programs makes it difficult to compare the results. A randomized controlled trial compared two parental education programs in pregnancy for first-time mothers^43^. There was no difference in the length of hospital stay in the random allocation of the two groups of mothers when measured on discharge before or after 48 hours after birth^43^. The two educational programs were similar regarding the topics, and both programs contained seven lessons, two-hours long before birth and one meeting six weeks after birth^43^. The lessons in the experimental group had more focus on pregnancy, birth, and early parenting as a life transition, where the control group prepared the parents for the topics as isolated events^43^. Furthermore, the experimental group used more group learning and fewer lectures by the facilitators than the control group^43^. Finally, the experimental group included activities for the parents to take home, where the control group did not have any activities to practice or discuss at home after the lessons^43^. The study found a significant difference between the parents' self-efficacy and perceived parenting knowledge in the two groups^43^. Thus indicating that the content and form of the parental education might also have a different impact on the outcomes of first-time mothers who are discharged within 24 hours. Therefore, we recommend that information about the content, form and setting of prenatal education is described in detail in future research. When healthcare professionals develop policies at the maternity wards regarding postnatal care for first-time parents, they must consider that first-time mothers under 25 years, with a low education level and little support at home, might differ in their need for information^36^. Only one study from 1989 examined how the mothers prioritized the information they received before discharge^36^. The study found a difference between first-time mothers and multiple mothers’ priorities of information that needs to be considered when the maternity wards organize the care from healthcare professionals after birth. However, more updated research of first-time parents needs for information before discharge is needed.

### The parents' satisfaction with early discharge after birth

Overall, the parents were satisfied with early discharge when the parents influenced the decision to be discharged early. The results of two studies in this scoping review emphasize the importance of support at home on the parents' satisfaction with early discharge^25,27^. The findings correlate with other studies that showed that support primarily from the husband and the women’s mothers positively influence a first-time mother’s confidence in infant care practices, breastfeeding and satisfaction^44,45^. However, the sample size in the two studies in this scoping review was small and more research is needed regarding how mothers and fathers best can be supported after early discharge. Furthermore, research of how healthcare providers can help prepare the father or family to support the mother is warranted in the future. The first-time fathers in the study by Feenstra et al.^26^ reported that the time from birth to discharge was stressful, revealing a potential gap in the literature to be explored in future research. Furthermore, more knowledge about the parents' characteristics that report having a positive versus a negative experience of early discharge is needed. Knowledge of any characteristics that might predict dissatisfaction with early discharge would aid in developing policies regarding early discharge at the maternity wards in the future. Finally, healthcare professionals should prioritize continuously care in the first hours after the birth to increase the parents' satisfaction and reduce stress after early discharge. The identified studies in this scoping review provided little knowledge of the parents' satisfaction immediately after birth and before discharge. Therefore, a gap in the literature has been identified, and more research about the parents' satisfaction with the time between birth and discharge is required in the future.

### Breastfeeding incidence after early discharge after birth

Discharge within 24 hours did not negatively influence breastfeeding incidence, compared to discharge after 24 hours. Furthermore, a home visit by a midwife on the third day was found to positively influence the mother’s experience of breastfeeding and the duration of breastfeeding. However, the different definitions of early discharge found in the literature can make the data ambiguous, e.g. according to breastfeeding, where we speculate that information differs for parents who are discharged within eight hours after birth compared to parents who are discharged 24 hours after birth. Little evidence was found regarding the impact early discharge has on breastfeeding incidence. Only one study by Quinn et al.^32^ found no difference in breastfeeding incidence between two groups discharged 24 hours after birth and 48 hours after birth; the study from 1997 included 101 firsttime mothers^32^. However, the study lacked a sample size calculation of the numbers of first-time mothers needed in the study to detect any significant difference between the two groups. More updated studies with more power are needed in future research. Only one study examined the mothers’ breastfeeding experience after early discharge^33^. The study found that breastfeeding support with emotional reassurance, promotion of positive self-efficacy and accessible breastfeeding support after early discharge, were important for the mothers’ breastfeeding experience and to sustain breastfeeding after early discharge^33^.

### Readmission after early discharge after birth

A large retrospective cohort study found that newborns of first-time parents who were discharged on the day of birth had a 21% higher risk of being readmitted than newborns of first-time parents who were discharged later^34^.

There was no information regarding follow-up strategies available in California at that time, making it difficult to generalize the results to other maternity wards. More updated studies are recommended in the future. No studies examined the severity of the newborns’ illness or duration of neonatal readmission when newborns were discharged early. Furthermore, no studies of maternal readmission were found, thus leaving a gap in the literature for future research.

### Early discharge after birth and maternity ‘blues’ and postpartum depression

This scoping review has identified a gap in the literature relating to maternity ‘blues’ or postpartum depression after early discharge. A study of 49 first-time mothers discharged within 48 hours after birth reported no difference in the mean depression score compared to those first-time mothers who were discharged on the third day after birth^46^. One-third of the first-time mothers left the hospital within 24 hours after birth^46^. However, the study did not clearly distinct between the mothers discharged before and after 24 hours after birth. Therefore, the study was not included in this scoping review. Postpartum depression can have profound implications in both the short- and long-term for the mother, the family, and the child’s development^47,48^. Therefore, research relating to the incidence and impact of maternity ‘blues’ and postpartum depression are essential in the future.

### Early discharge after birth and the fathers or co-parents

In this scoping review, most of the identified literature focused on the mother and the newborn. Very little evidence was found focusing on the father or co-parent. Therefore, there is a gap in the literature regarding the fathers or coparents experience of early discharge. Furthermore, we have little knowledge of what information the father or co-parent needs before taking care of the new family at home, which needs to be addressed in future research. Finally, there were no studies of the fathers or co-parents incidence of depression after discharge within 24 hours after birth.

### Systematic reviews of early discharge after birth

Two systematic reviews in 2010 and 2017 compared early discharge with the standard length of hospital stay after the birth of healthy mothers and newborns^14,15^. The first review by Benahmed et al.^15^ found no statistical difference between early discharge in neonatal or maternal outcomes. The studies included in the review were rated as of low to very low evidence^15^. This review did not examine the results separately for first-time mothers and multiple mothers, making it difficult to transfer the results to first-time mothers. The other review from the Cochrane Collaboration found no statistical difference in neonatal or maternal readmission or breastfeeding incidence^14^. Moreover, no difference in postpartum depression was found in the Cochrane review regarding discharge time. The authors of the Cochrane review had planned subgroup analysis of firsttime mothers compared to multiple mothers but was unable to perform the sub-analysis. Furthermore, the definition of early discharge in the two reviews varied from 6 to 96 hours. Moreover, the length of the standard stay after birth also varied from 48 hours to four or more days from birth^14,15^. Thus, the above mentioned make it difficult to transfer the results to first-time parents discharged within 24 hours.

### Strengths and limitations

This scoping review is strengthened by the rigorous methods based on prespecified criteria in protocols for scoping reviews. However, our review was limited by screening only articles available in English or Scandinavian languages. In addition, many articles were excluded from this scoping review because there was a lack of clear distinction between primiparas and multiparas, vaginal birth or caesarean section, and discharge before or after 24 hours following the birth. Therefore, there may be evidence relevant to the topic of this scoping review that has not been included. Due to the heterogeneity in methodology, terminology and assessment procedures used in the included articles, the generalization of study results is limited. Findings of this review highlight the importance of identifying factors and outcomes related to early discharge.

## CONCLUSIONS

Based on the identified evidence, the healthcare professionals need to consider how to address the problem if the parents lack social support at home to avoid adverse outcomes of early discharge when developing an early discharge policy. Furthermore, the policy should specify the potential need for more information when the mothers are under 25 years or have low educational level. The maternity wards should also prioritize for the midwives to have time to be with the parents in the first hours after birth to avoid the parents' dissatisfaction and feelings of stress before early discharge after birth. The healthcare professionals should ensure that the parents feel ready for discharge and that they have some influence on the time for discharge. Healthcare professionals should be available around the clock for firsttime parents to increase the parents' sense of security and give breastfeeding support. Finally, the early discharge policy should contain a systematic follow-up strategy. In this scoping review, we found that daily contact with healthcare professionals in the first week after discharge, one visit by the midwife on the third day after discharge supplemented with written information about the health of the mother and newborn, might be enough to make first-time parents feel secure and to support breastfeeding when first-time parents are discharged within 24 hours after birth.

This scoping review has documented that there is limited evidence of the outcome and factors that influence the outcomes for first-time parents who are discharged within 24 hours after birth. More updated research is needed regarding breastfeeding incidence, the parents need for information before early discharge, and neonatal readmission. In addition, numerous gaps in the literature have been identified: 1) the fathers or co-parents experience of early discharge, 2) the fathers or co-parents need for information before early discharge, 3) the duration and severity of neonatal readmission, 4) postpartum depression of both parents, 5) a gap regarding a detailed description of the content and form of parental education in pregnancy is missing in the existing literature, and 6) the number of home visits needed for the parents to feel safe being discharged early requires more research. It is recommended that future research of the home visit after discharge is described in detail. Future studies should implement study designs that lead to stronger evidence, a clear definition of early discharge and uncomplicated birth using standardized descriptions of criteria, context, and intervention.

## Data Availability

Data sharing is not applicable to this article as no new data were created.
